# Insights into Pathogenesis of Trachoma

**DOI:** 10.3390/microorganisms12081544

**Published:** 2024-07-28

**Authors:** Panagiotis Toumasis, Georgia Vrioni, Ioannis T. Tsinopoulos, Maria Exindari, George Samonis

**Affiliations:** 1 MSc in Ocular Surgery, School of Medicine, Aristotle University of Thessaloniki, 541 24 Thessaloniki, Greece; toumasispanagis@gmail.com (P.T.); itsinop@auth.gr (I.T.T.); mexidari@auth.gr (M.E.); 2 Department of Microbiology, School of Medicine, National and Kapodistrian University of Athens, 115 25 Athens, Greece; gvrioni@med.uoa.gr; 3 Second Department of Ophthalmology, School of Medicine, Aristotle University of Thessaloniki, Papageorgiou General Hospital, 564 29 Thessaloniki, Greece; 4 Department of Microbiology, School of Medicine, Aristotle University of Thessaloniki, 541 24 Thessaloniki, Greece; 5 School of Medicine, University of Crete, 715 00 Heraklion, Greece; 6 Metropolitan Hospital, 185 47 Piraeus, Greece

**Keywords:** trachoma, chlamydia trachomatis, transmission, pathogenesis, trachomatous scarring, trachomatous trichiasis

## Abstract

Trachoma is the most common infectious cause of blindness worldwide. This review investigates the pathogenesis of trachoma, focusing on its causative agent, transmission pathways, disease progression, and immune responses. Trachoma is caused by serovars A–C of the bacterium Chlamydia trachomatis (Ct). Transmission occurs through direct or indirect exchanges of ocular and nasal secretions, especially in regions with poor hygiene and overcrowded living conditions. The disease is initiated in early childhood by repeated infection of the ocular surface by Ct. This triggers recurrent chronic inflammatory episodes, leading to the development of conjunctival scarring and potentially to trichiasis, corneal opacity, and visual impairment. Exploring the pathogenesis of trachoma not only unveils the intricate pathways and mechanisms underlying this devastating eye disease but also underscores the multifaceted dimensions that must be considered in its management.

## 1. Introduction

Trachoma, an enduring relentless scourge on human populations, persistently exacts a profound toll on public health, standing as the leading infectious cause of blindness globally [1,2]. Trachoma is a type of chronic keratoconjunctivitis caused by the bacterium Chlamydia trachomatis (Ct). Nowadays, trachoma is endemic in much of sub-Saharan Africa, areas of the Middle East crescent, and pockets of Asia and South America, being a public health problem in a total of 40 countries [3]. The World Health Organization (WHO) has included trachoma in a comprehensive portfolio of neglected tropical diseases earmarked for targeted elimination [4]. The persistent nature of trachoma, coupled with its potential to cause irreversible harm, accentuates the urgency of concerted efforts to combat and eradicate this infectious affliction.

Trachoma has been extensively documented, holding a significant place in historical texts. The earliest evidence comes from China around 2600 B.C., while the initial detailed account of the disease dates back to 1500 B.C. in the Ebers Papyrus—a compilation of medical prescriptions discovered in Egypt [5]. Despite being such an ancient ailment, our understanding of trachoma continues to evolve, and its impact persists in contemporary times, especially in impoverished and overcrowded communities worldwide.

This narrative review aims to delve into the pathogenesis of trachoma, analyzing the causative agent, transmission pathways, the natural history of the disease, and the immune responses elicited within the human organism, serving as a valuable knowledge repository. Also, this review endeavors to contribute to the ongoing discourse on trachoma, fostering a deeper understanding of its complexities and paving the way for informed discussions on preventive and therapeutic interventions, encouraging continued efforts towards the ultimate goal of totally eliminating this particularly disabling disease.

## 2. The Causative Agent

Ct is a Gram-negative, anaerobic, intracellular obligate eubacterium that falls within the genus Chlamydia and, more broadly, the Chlamydiaceae family [6]. Ct infects humans at the epithelial layer of mucosal surfaces. Ct is classified into multiple serovars, which are serologically variant strains distinguished on the basis of specific epitopes of the major outer membrane protein (MOMP) [7,8]. A single-copy chromosomal gene, *ompA*, encodes this protein, which constitutes more than half of the cell wall mass and plays a role in adhering to the surface of host cells [8]. There are 15 major Ct serovars [9]. These serovars display well-documented and unique tissue tropism. Specifically, serovars A–C are recognized as the causative agents of trachoma [9]. Except for serovar B variants, which are linked to a minimal occurrence of urogenital disease, the serovars associated with trachoma are seldom found in the genital tract [10]. Serovars D–K are significant contributors to urogenital tract infections worldwide, such as urethritis, cervicitis, proctitis, epididymitis, and pelvic inflammatory disease, but they are not associated with trachoma [9]. Yet, serovars D–K have the potential to induce ocular infections: neonatal conjunctivitis (Ct is the most common cause of neonatal conjunctivitis, “ophthalmia neonatorum”) typically presents during the first 5 to 14 days of life, acquired during delivery, and adult inclusion conjunctivitis, which is considered a sexually transmitted infection (STI), is transmitted through the hand-to-eye spread of infected genital secretions, primarily affecting sexually active individuals [11,12]. Both serovars A–C and serovars D–K can affect the eyes, but the impact varies significantly. While the serovars A–C induce trachoma, a chronic ocular infection with a high risk of blindness, serovars D–K result in inclusion conjunctivitis, a transient and self-limiting infection of the conjunctival mucous membrane without severe complications. Serovars L1–L3 cause an invasive infection of the lymph nodes near the genitals, called lymphogranuloma venereum (LGV) [9]. The serovars of Ct are grouped into three main human biovars: serovars A–C have been grouped into the trachoma biovar, serovars D–K have been grouped into the genital tract biovar, and serovars L1–L3 constitute the LGV biovar [9] (Table 1).

The diverse manifestations of Ct infections are attributed to inactivating mutations found in the genomic sequences of the tryptophan synthase operon (trpRBA), located within a region known as the plasticity zone (PZ) [13,14,15]. Ocular serovars A, Ba, and C have mutations in the TrpA gene, which codes the α subunits of tryptophan synthase, resulting in the synthesis of a truncated, enzymatically inactive TrpA. In contrast, all urogenital serovars (D–K) encode a full-length, active TrpA [15]. Also, the infrequent genital isolates of serovar B, a serovariant typically linked to trachoma, exhibit intact *trp* genes responsible for encoding a functional TrpBA complex—a feature exclusive to genital serovars [15]. A potential explanation could be that ocular serovars might have the ability to obtain tryptophan from a source within the conjunctival sac or infected cells, whereas this source is not accessible to pathogens in the urogenital tract.

Ct has a life cycle consisting of two morphologically distinct forms: the elementary body (EB), which is the small, metabolically inactive extracellular form of the microorganism in which it moves between host cells, namely human epithelial cells, and the reticulate body (RB), the larger, metabolically active, intracellular stage [7,9,16]. The ΕΒ possesses a sturdy cell wall, enabling it to endure adverse conditions. The chlamydial developmental cycle begins when the infectious EB attaches to the host cell and enters it through receptor-mediated endocytosis. The EB, surrounded by a vacuole formed by the host cell membrane and chlamydial proteins, develops into a peri-nuclear inclusion. Once engulfed within the inclusion, the EB undergoes a transformation into the RB form which replicates by binary fission over the following 30 to 72 h. The massive number of RBs then proceed to transition back to EBs with a condensation of nuclear material and an overall reduction in size, before being released into the environment through the lysis of the host cell. These new ΕΒs then attach to new host cells.

## 3. Transmission

**Figure 1:**
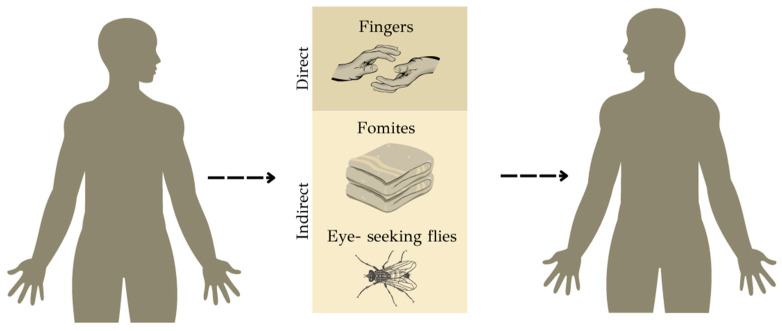
Routes of trachoma transmission−direct (fingers between humans) and indirect (fomites and eye-seeking flies) means of Ct spread.

Trachoma spreads through the exchange of ocular and nasal secretions, both directly and indirectly between individuals (Figure 1). Direct transmission occurs through face-to-face activities, like face touching. In indirect transmission, fomites play a crucial role, wherein contaminated objects such as bedding or towels act as intermediaries in passing on the infection [17,18]. An experimental study has shown that Ct can remain viable on plastic, cotton cloth, and skin for over 24 h [17]. An intriguing aspect of the transmission dynamics is the involvement of eye-seeking flies, particularly *Musca sorbens*, which selectively gather protein from human exudates to support their egg production [19]. There is still uncertainty about whether Ct infects and replicates within vector fly species. However, a recent study, where flies from the same genus as Musca sorbens were fed with Ct, confirmed the presence of viable EBs in fly crops up to 24 h post feeding and the presence of Ct DNA up to 48 h post feeding [20]. Furthermore, it demonstrated that these flies can regurgitate and transmit Ct at their next feeding [20]. Also, a recent study has demonstrated that managing fly populations with insecticides decreases the incidence of active trachoma disease [21]. A combination of these modes of transmission probably functions in most environments, with their relative importance varying between different communities and between members of a community.

**Figure 2:**
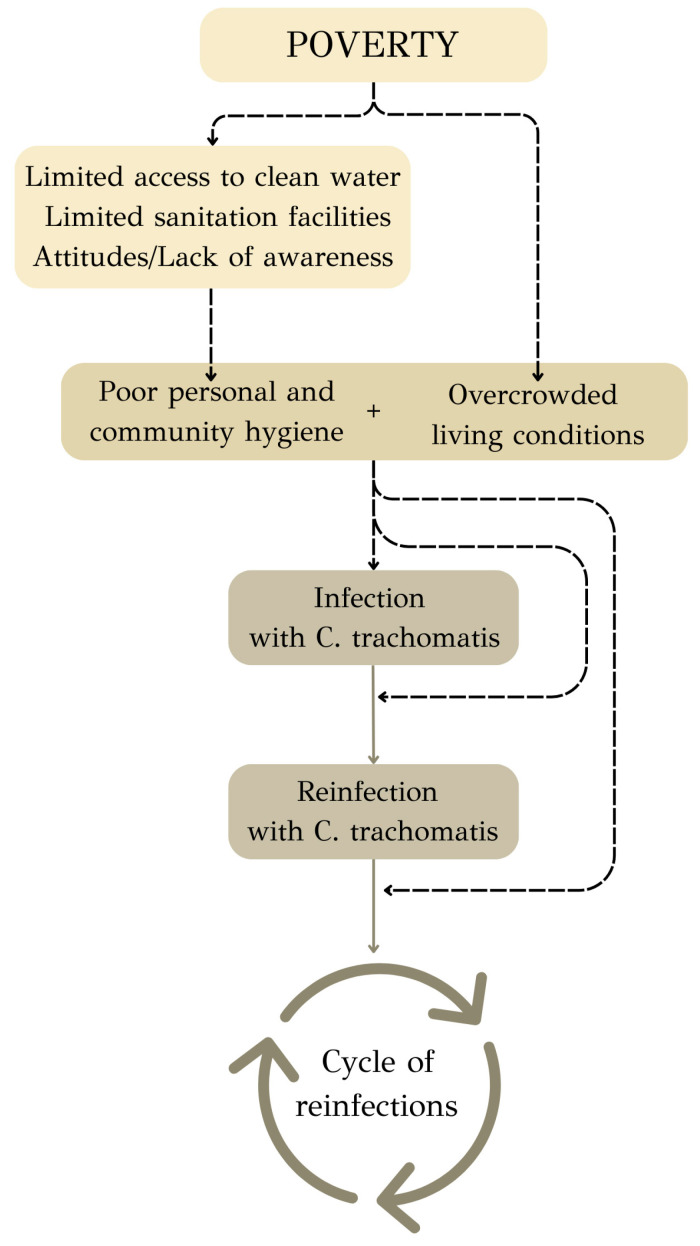
Interplay of factors contributing to trachoma.

In endemic areas, specific factors contribute to the swift and sustained transmission of the disease (Figure 2). The transmission of trachoma is amplified by the lack of proper personal and community hygiene [22]. This deficiency is often rooted in several interconnected factors. Limited access to clean water may pose a critical challenge, as it hinders individuals’ ability to maintain basic personal hygiene practices, such as regular handwashing and face cleaning. However, water storage capacity and the way that water is utilized are more essential for facial cleanliness [23]. In communities where sanitation facilities such as toilets and sewage systems are inadequate or unavailable, the disposal of waste becomes a persistent issue, further contributing to the compromised hygiene of the community [22]. Moreover, attitudes and a lack of awareness play a pivotal role in shaping hygiene behaviors [24]. In some cases, communities may not fully realize the importance of hygiene practices in preventing trachoma transmission. Additionally, overcrowded living conditions further facilitate the contagious nature of trachoma [25]. In such environments, where individuals not only live in close proximity but also share sleeping quarters, the risk of direct contact and the exchange of infectious particles increases substantially.

## 4. Natural History and Clinical Features of Trachoma

Trachoma can be conceptualized as progressing in two distinct but overlapping stages. Ιn the first stage, repeated infections with ocular strains of Ct induce recurrent episodes of chronic conjunctival inflammation, which results in the manifestation of the clinical signs of what is referred to as “active trachoma” [26]. A key clinical sign of active trachoma is follicular conjunctivitis, most prominently of the upper tarsal conjunctiva. Follicular conjunctivitis is identified by the presence of follicles, clusters of lymphoid tissue located beneath the epithelial layer of the tarsal conjunctiva. In some cases, the inflammation can be more intense, with severe papillary hypertrophy, which refers to the diffuse infiltration, edema, and enlargement of vascular tufts that can obscure deep tarsal vessels [26].

**Figure 3:**
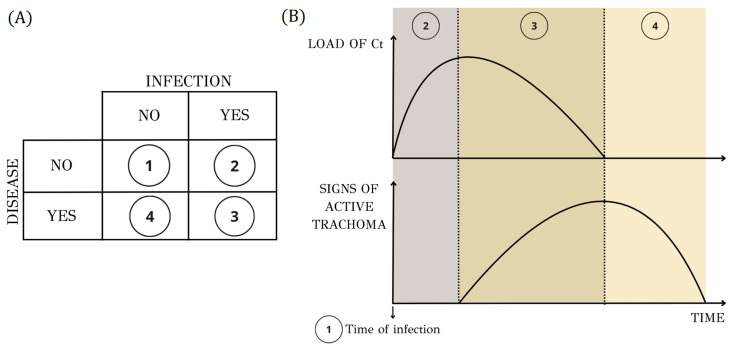
Relationship between the appearance of clinical signs of active trachoma and the load of the causative agent (Ct). (**A**) Quadrilateral table of the four states of infection and disease with states indexed from 1 to 4. (**B**) Diagram illustrating the kinetics of trachoma, highlighting that active inflammatory disease persists for a period even after the load of Ct becomes undetectable. (1) Time of infection—the load of Ct increases. (2) Incubation period—infection, but no clinical signs are observed. (3) Evident disease—clinical signs have become apparent. (4) Recovery period—infection cleared, and clinical signs persist but gradually resolve. (Disclaimer: the duration of each period is not representative).

The correlation between active disease and infection in trachoma is intricate, revealing a disparity between clinical manifestations of active trachoma and the identification of Ct [27] (Figure 3). After infection with Ct, there is a short ‘preclinical’ phase lasting a few days before the onset of inflammation symptoms, known as the incubation period. A mathematical model describing the natural history of trachoma infection and disease estimated the median incubation period at 17 days (95% CI: 11–28) [28]. This period is followed by a phase of evident disease where the infectious agent and clinical signs coexist. Finally, there is a recovery period during which the human immune response acts in clearing the infectious agent or reducing it to undetectable levels, while the clinical signs of active trachoma persist and gradually diminish. Clinical manifestations endure for an uncertain yet presumably prolonged duration following the clearance of the infectious agent, unless there is a subsequent encounter with the chlamydial antigen. The mathematical model mentioned before estimated that the duration of the disease is longer than that of the infection, with a median of 21 weeks (range: 15–32 weeks) compared to 17 weeks (range: 12–24 weeks) [28]. Additionally, the estimated median duration of inflammation following the infection was 5 weeks (range: 3–8 weeks) [28].

Persistent re-infections contribute to a chronic cicatricial process [2]. With time, inflammation-induced tissue damage results in scarring of the eyelid, marking the onset of the second stage of the disease. The recovery of the inflamed conjunctiva involves some residual conjunctival scarring. A mathematical model describing the transmission of Ct and its progression to cicatricial disease estimated a threshold, forecasting that more than 100 infections during an individual’s lifetime are needed to generate trachomatous scarring [29]. Conjunctival scarring encompasses anything from a few linear or stellate scars to fibrous bands that shorten the fornix and create symblepharons (adhesions of the palpebral conjunctiva to the bulbar conjunctiva). Chronic conjunctival inflammation is commonly observed in adults who have developed conjunctival scarring [30,31,32]. This subsequent stage of conjunctival inflammation typically lacks follicular characteristics, with a rare detection of Ct infection. Instead, it is marked by a significant innate epithelial immune response. Factors such as non-chlamydial infections, along with the dryness and irritation of the scarred conjunctiva, may also play a role in promoting inflammation and advancing the progression in the presence of established scarring [33].

As the scar tissue contracts, it causes the eyelids to turn inward, resulting in entropion. The condition known as trichiasis occurs when eyelashes make contact with the eye. In the case of trachoma, trichiasis often originates from entropion, although this is not a strict rule. Many patients with trachomatous trichiasis have minimal or no entropion and trichiasis arises from misdirected lashes in a normal position (aberrant lashes) or lashes growing from abnormal locations (metaplastic lashes) [34]. The same mathematical model mentioned before predicts that more than 150 infections are necessary for the development of trachomatous trichiasis [29]. The potential harm resulting from the cornea being scratched by inward-turned eyelashes extends beyond immediate discomfort. Continuous friction from misdirected eyelashes injures the corneal surface, leading to corneal opacification. This, in turn, can result in impaired vision and, in severe cases, blindness. Untreated trichiasis can lead to corneal opacity and vision loss in as little as a year in about one-third of individuals [35]. It is essential to emphasize that corneal opacification and blindness likely result from traumatic damage caused by both trichiasis and secondary infections from other pathogens, such as bacteria or fungi [36,37].

**Figure 4:**
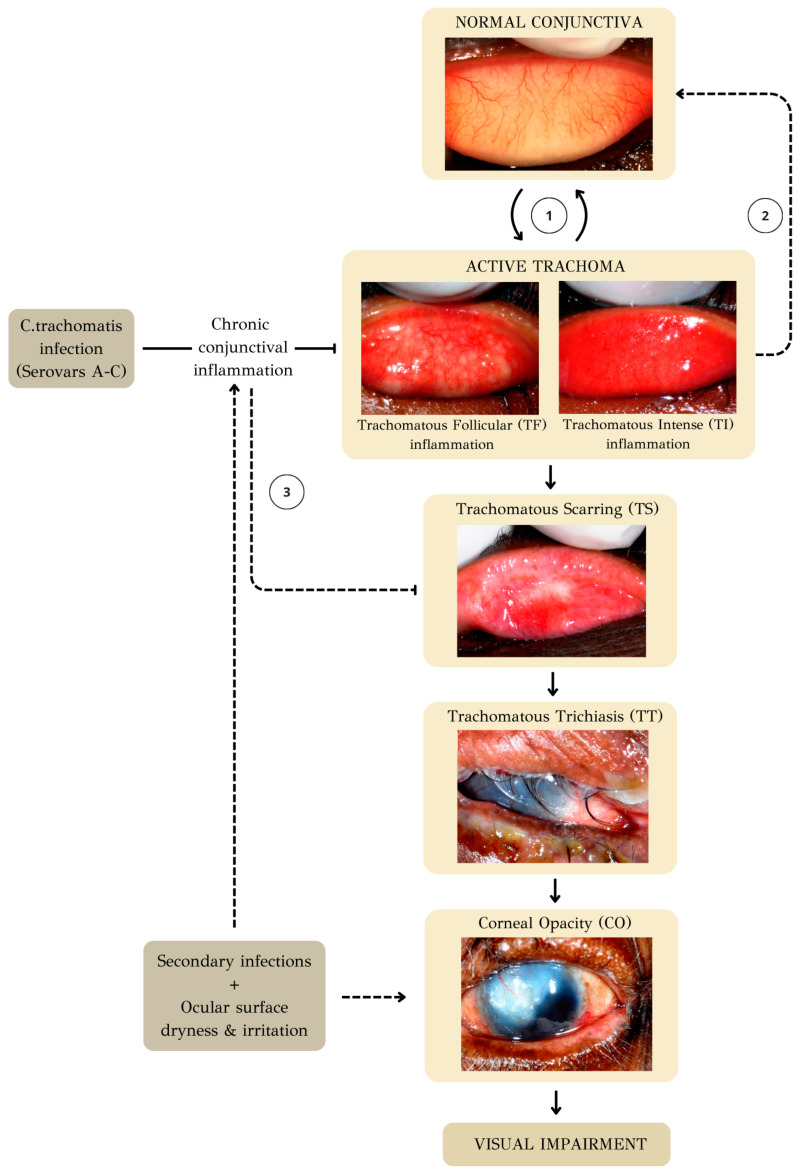
The natural history of trachoma. Persistent re-infections contribute to the chronic cicatricial process (1). The recovery of the inflamed conjunctiva (2) involves some residual conjunctival scarring (3). [The clinical images are adapted from Ref. [30], CC BY 4.0 (https://creativecommons.org/licenses/by/4.0/ accessed on 26 May 2024)].

The clinical features mentioned above are classified using the simplified WHO trachoma grading system [2,38]. This simplified grading system, suggested by the WHO in 1987 and amended in 2018, is specifically designed to enable a quick evaluation of the prevalence and severity of the disease within a population. It is as follows:

1. Trachomatous Follicular (TF) inflammation: The stage of active trachoma with predominantly follicular inflammation. Diagnosing this stage requires the presence of at least five or more follicles on the upper tarsal conjunctiva, each measuring 0.5 mm or more in diameter.
2. Trachomatous Intense (TI) inflammation: This stage of active trachoma is identified when significant inflammatory thickening of the upper tarsal conjunctiva obscures more than half of the normal deep tarsal vessels. The tarsal conjunctiva displays a reddish hue, a coarse texture, and increased thickness—the results of papillary hypertrophy. It should be mentioned that the key sign for the diagnosis of TI is thickened, velvety edematous conjunctiva, not redness.
3. Trachomatous Scarring (TS): This stage is identified through the detection of scarring in the tarsal conjunctiva, which is apparent as white lines, bands, or sheets (fibrosis) in the tarsal conjunctiva. Typically, the scars exhibit a shiny and fibrous appearance, featuring straight, angular, or feathered edges. Scarring, particularly diffuse fibrosis, might obscure the blood vessels in the tarsal area, but it is crucial not to mistake this for diffuse inflammatory thickening.
4. Trachomatous Trichiasis (TT): This stage is identified when at least one eyelash from the upper eyelid rubs on the eyeball, or there is evidence of recent removal of in-turned eyelashes from the upper eyelid.
5. Corneal Opacity (CO): This stage is recognized when there is a clearly visible corneal opacity covering the pupil. The corneal opacity is so dense that at least part of the pupil margin is blurred when viewed through the opacity. This definition aims to identify corneal opacities that result in substantial visual impairment (Figure 4).

**Figure 5:**
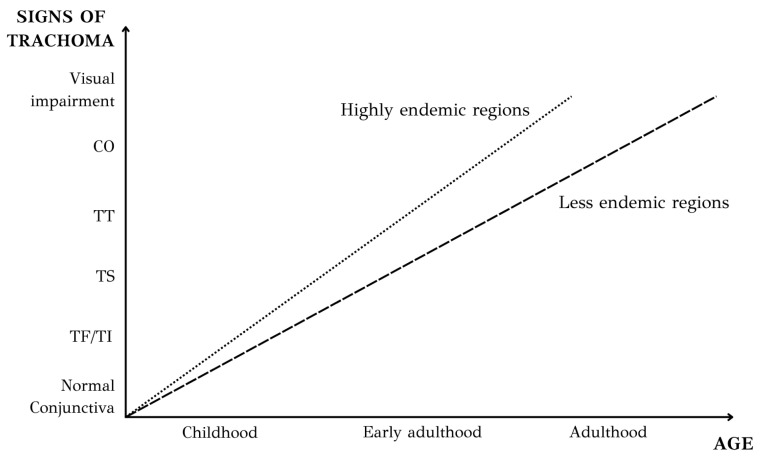
Diagram illustrating the correlation of clinical signs of trachoma with age. In highly endemic regions, blinding complications of trachoma emerge at an earlier age. (TF/TI: Trachomatous Follicular/Intense inflammation, TS: Trachomatous Scarring, TT: Trachomatous Trichiasis, CO: Corneal Opacity).

The signs of trachoma exhibit a significant correlation with age (Figure 5). In a typical endemic setting, repeated chlamydial infection of the conjunctiva begins early in life. The prevalence of active trachoma (TF/TI) peaks in preschool children [39,40,41]. A recent meta-analysis focused on the pediatric population showed that the key factors associated with active trachoma among children are the absence of latrines, children’s unclean faces, and the lack of reported soap use for washing [42]. The occurrence of active trachoma diminishes as individuals age, with only a small number of adults displaying signs of active disease and even fewer presenting proof of infection [28,39,43,44]. On the contrary, there is an escalation in the prevalence of trachomatous conjunctival scarring as individuals age, underscoring the cumulative nature of the damage. The long-term sequelae of trachoma infection in adults relates to their exposure to active trachoma during the first months of life. The scarring sequelae of trachoma typically emerge in adulthood, generally starting around the third decade of life. However, in areas with a higher prevalence of severe disease, these effects may manifest earlier. Furthermore, there is a correlation between age and the duration of active disease but not with the duration of infection [28]. As individuals age, there is a statistically significant decline in the duration of active disease. Although a reduction in the duration of infection is observed, this decrease is non-significant.

Trachoma exhibits a notable correlation with gender. Male and female children tend to be equally affected by active trachoma. On the other hand, studies consistently highlight that females engaged in childcare tasks present a higher prevalence of active disease compared to those not involved in caregiving duties [39,45]. As a logical consequence, a higher prevalence of blinding complications of trachoma is observed among women compared to men, supporting the hypothesis of a multifaceted interplay between biological, social, and cultural factors [39,46,47].

## 5. Histopathology and Immunopathology

The conjunctiva, a thin and transparent mucous membrane covering the front part of the eye and lining the inner surface of the eyelids, exhibits a layered histological arrangement [48]. Externally, there is a stratified non-keratinizing squamous epithelium of three to five cell layers, with interspersed goblet cells that produce important mucins for the tear film that covers the epithelium. Beneath the epithelium lies the stroma, which consists of superficial lymphoid and fibrous tissue.

Tissue changes in active follicular trachoma begin with the dilation of capillaries and small venules, accompanied by the infiltration of inflammatory cells (lymphocytes, macrophages, neutrophils, dendritic cells and plasma cells) [49]. B cells are surrounded by a mantle of T cells, forming lymphoid follicles [49]. Once they reach a significant size, lymphoid follicles alter the shape of the covering epithelium and become observable in the inverted tarsal conjunctiva. Also, there is a generalized increase in the amounts of types I, III, and IV collagen, which typically present in the stroma, and a deposition of new type V collagen [50]. Trachomatous scarring involves epithelial atrophy (epithelial cells become thinned), a decrease in tear film volume with altered composition, and a depletion of goblet cells [51]. The stroma loses its regular architecture and transforms into a dense scar composed of type V collagen, while these fibers firmly adhere to the tarsal plate, resulting in distortion [51,52]. Immunohistochemical staining in conjunctival biopsy samples from individuals with trachomatous scarring showed the infiltration of immune cells with a phenotype suggestive of natural killer (NK) cells [53]. Also, contrary to our expectations based on previous studies, a significant portion of inflammatory cells in this study were found to be CD45-negative [53]. It was anticipated that the majority, if not all, of these inflammatory cells would exhibit CD45 expression, as CD45 is typically present on nearly all nucleated cells of hematopoietic origin. Regarding trachomatous trichiasis conjunctival tissue, it exhibits notably elevated expression levels of proteins S100A7 and CTGF in the epithelium, along with a significantly increased expression of IL-1β in the subepithelial tissue [54].

Diseases caused by chlamydia are based on intense and chronic inflammation elicited and maintained by re-infection or persistent infection. Ct infection triggers an innate immune response. Ct-infected epithelial cells directly initiate this process by detecting the presence of the pathogen through their pattern recognition receptors [55,56]. Findings from both in vitro and in vivo studies revealed a significant proinflammatory reaction, marked by the production of several chemokines and cytokines [56,57,58,59]. The former act as chemoattractants, organizing the migration of immune cells towards the site of infection, and the latter are involved in the up-regulation of inflammatory reactions. Another suggestion, though with little evidence from studies on humans, is that the disease is mediated by antigen-dependent delayed-type hypersensitivity or autoimmunity [30,55,60].

The infection can resolve or progress to eyelid scarring. The resolution of Ct is believed to rely on the presence of interferon gamma (IFNγ) derived from an immune response mediated by CD4+ T helper 1 (TH1) cells. Examinations of gene expression in children residing in trachoma-endemic regions reveal a significant rise in the conjunctival expression of IFNγ and factors associated with TH1 cells, such as IL-12B and indoleamine-2,3-dioxygenase (IDO1), along with increased activity in NK cell pathways marked by NCR1, CD56, and CD247 [57,58,59]. In cases of active trachoma without detectable Ct, levels of IFNγ are not notably elevated, suggesting a swift regulatory response once the infection has cleared. Infection and active trachoma are associated with a significant TH17 response, which is characterized by heightened levels of IL-17, IL-21, and IL-22 [59]. Also, some evidence suggests that TH17–IL-17 responses could potentially aggravate the inflammatory and scarring reaction [61].

The primary clinical indicator associated with the formation of scarring is intense conjunctival inflammation, which correlates with a heightened expression of various pro-inflammatory factors [59]. Additionally, studies have found that both active and scarring trachoma show increased amounts of matrix metalloproteinases (MMPs) 7, 9, and 12 [31,57,59,62]. MMPs are a family of extracellular matrix (ECM) remodeling endopeptidases that have the capacity to degrade almost every component of the ECM, promoting the contraction of scars. A single-nucleotide polymorphism (SNP) in the catalytic domain of MMP-9 possibly leads to reduced function and is linked to a decreased likelihood of scarring [63]. Moreover, active trachoma and scarring have been correlated with the heightened expression of numerous fibrogenic growth factors.

## 6. Conclusions

Exploring the pathophysiology of trachoma not only unveils the intricate pathways and mechanisms underlying this devastating eye disease but also underscores the multifaceted dimensions that must be considered in its management.

The first crucial dimension revolves around prevention strategies based on the transmission pathways and the complex interplay of risk factors contributing to the disease. The initial pivotal aspect involves prevention strategies rooted in understanding the transmission pathways and the intricate interplay of risk factors contributing to trachoma. To interrupt the cycle of trachoma transmission, it is imperative to promote hygiene by educating communities on proper face washing. Additionally, environmental improvement is essential, necessitating the implementation of water and sanitation infrastructure projects. Socio-economic development plays a crucial role, requiring a focus on poverty alleviation strategies, the economic empowerment of communities, and enhancements in overall living conditions. Furthermore, it is crucial to emphasize surveillance and monitoring through the implementation of robust systems. This approach aims to detect populations at risk and areas that may be experiencing great numbers of trachoma cases, ensuring timely and targeted preventive and therapeutic measures.

Another crucial dimension is the mismatch noted between infection and disease. This fact holds significant implications for trachoma programs. Making antibiotic treatment decisions based solely on observable conjunctival inflammation of at least moderate intensity, aimed at individual targets, would overlook many individuals with mild clinical responses who are still infected and require treatment.

The WHO-led Global Alliance for the Elimination of Trachoma recommends the implementation of the SAFE strategy which tackles the disease at different stages: Surgery to correct trichiasis, Antibiotics to treat chlamydial infection, and Facial cleanliness as well as Environmental improvements to suppress the transmission of infection. The strides taken by the WHO in curbing the prevalence of trachoma represent a commendable achievement, reflecting a commitment to global public health. However, it is crucial to recognize that the battle against this eye disease is far from over.

Trachoma’s impact extends far beyond the field of medicine, reaching into the social, economic, and educational domains of affected communities. Addressing each dimension with precision and synergy can provide hope that trachoma can be effectively limited, ultimately alleviating its impact on the social burden and improving global health.

## Figures and Tables

**Table 1:** Chlamydia trachomatis–biovars, serovars, and associated diseases.

Biovars	Serovars	Main Diseases
Trachoma	A–C *	Trachoma
Genital	D–K	Neonatal conjunctivitis Adult inclusion conjunctivitis Pneumonia (neonates)
LGV	L1–L3	LGV

LGV = Lymphogranuloma venereum; * B/Ba: linked to both, ocular and genital tract infection.

## Data Availability

Data are contained within the article.
